# Comprehensive positional and morphological assessments of the temporomandibular joint in adolescents with skeletal Class III malocclusion: a retrospective CBCT study

**DOI:** 10.1186/s12903-023-02788-4

**Published:** 2023-02-07

**Authors:** Yanxi Chen, Lingfeng Li, Ying Li, Nan Luo, Hongwei Dai, Jianping Zhou

**Affiliations:** 1 Stomatological Hospital of Chongqing Medical University, 426, Songshi North Road, Chongqing, China; 2 Chongqing Key Laboratory of Oral Diseases and Biomedical Sciences, Chongqing, China; 3 Chongqing Municipal Key Laboratory of Oral Biomedical Engineering of Higher Education, Chongqing, China

**Keywords:** Temporomandibular joint, Skeletal Class III malocclusion, Adolescents, Cone-beam computed tomography

## Abstract

**Background:**

Condyle-fossa relationships in adolescents with skeletal Class III malocclusion remain unclear. Therefore, this study used cone-beam computed tomography (CBCT) to evaluate the position and morphology of the temporomandibular joint (TMJ) in adolescents with skeletal Class III malocclusion.

**Methods:**

In this cross-sectional retrospective study, CBCT images from 90 adolescents with skeletal Class III malocclusion and 30 controls were analysed. Adolescents with skeletal Class III malocclusion were divided into different groups based on (1) sex (male and female), (2) sides (right and left), (3) age (early, middle, and late adolescence), and (4) vertical skeletal patterns (hyperdivergent, normodivergent, and hypodivergent). Morphology of the condyle and fossa as well as condylar position, was compared among groups. Data were collected and submitted for statistical analysis. This study adheres to STROBE guidelines.

**Results:**

Regarding the intergroup comparisons, there were significant differences in TMJ position and morphology between the skeletal Class III malocclusion with different vertical skeletal patterns and control groups (*P* < 0.05). Within groups, condyle-fossa relationships differed significantly according to sex, age, and vertical skeletal patterns (*P* < 0.05); however, the mean values were not statistically different between left and right sides in adolescents with skeletal Class III malocclusion.

**Conclusions:**

Our findings can be used clinically and radiographically to evaluate the condyle and glenoid fossa features in adolescents with skeletal Class III malocclusion, providing a basis for better TMD diagnosis and orthodontic treatment.

**Supplementary Information:**

The online version contains supplementary material available at 10.1186/s12903-023-02788-4.

## Background

Skeletal Class III malocclusion is a relatively common occlusal problem characterised by a sagittal deformity in which the mandible is positioned mesial to the maxilla. There are wide variations in the reported prevalence of skeletal Class III malocclusions, ranging from 4.2 to 31.4%, both between and within populations [1, 2]. Genetic variants, oral digit habits, abnormal tongue and mandibular position, nasal blockage, trauma, and other factors are possible aetiologies of skeletal Class III malocclusion [3]. It may have a long-term effect on the growth and development of teeth and the skeletal bases that support them, altering proper occlusal function in these patients due to a sagittal deficiency of the maxilla in relation to the mandible.

In individuals with skeletal Class III malocclusion, the anatomical structure between the upper and lower dentitions is disrupted, leading to abnormal jaw movements. In addition, this impaired maxillomandibular relationship can impact multiple aspects of mastication, including muscle activity, occlusion, and jaw movement during chewing, which place abnormal pressure on the orofacial structures. The temporomandibular joint (TMJ) undergoes long-lasting remodelling due to the adaptive response to mechanical stress, leading to positional and morphological changes in the TMJ, including the cartilage, mandibular fossa, and condyle [4, 5]. Therefore, it is reasonable to suspect that skeletal Class III malocclusion would affect the position and configuration of the TMJ.

The condyle-fossa relationship is related to functional anatomy. The effects of skeletal Class III malocclusion on the condyle-fossa relationship vary from none to major. Arieta-Miranda et al*.* [6] concluded that individuals with skeletal Class III malocclusion had a smaller upper distance of the condyle than those with skeletal Class I malocclusion. Paknahad et al*.* [7], however, demonstrated no spatial differences in the position of the condyles between the control and skeletal Class III malocclusion groups. Katsavrias et al*.* [8] observed that participants with skeletal Class III malocclusion had a wider and shallower articular fossa, but Seren et al*.* [9] found that the width of the glenoid fossa (WF) was smaller in participants with skeletal Class III malocclusion than in those with skeletal Class I malocclusion. Recently, Chae et al*.* [10] reported no substantial variations in the structures and position of the TMJ between individuals with skeletal Class III malocclusion and controls. Given these inconsistent findings, additional investigations are required to clarify the effects of skeletal Class III malocclusion on TMJ morphology and position. Notably, the positional and structural characteristics of the TMJ may be related to side (left and right) [10, 11], sex [12, 13], age [11, 14], and skeletal vertical patterns [6, 8, 15–17]. However, the relevant quantitative investigations on TMJ components in adolescents with skeletal Class III malocclusion remain scarce.

Cone-beam computed tomography (CBCT) has gained popularity as a diagnostic tool for various TMJ diseases over the past few years. Some researchers have demonstrated the superiority of this imaging modality over traditional radiographic modalities in the assessment of the TMJ region [18]. Accordingly, this study aimed to use CBCT to identify the positional and morphological properties of the TMJ in adolescents with skeletal Class III malocclusion considering side, sex, age, and vertical skeletal patterns.

## Methods and materials

### Participants and eligibility criteria

The Research Ethical Committee of the Stomatological Hospital of Chongqing Medical University approved this cross-sectional CBCT-based study (No. 2021-026). The sample size was calculated using G*Power software (Version 3.1, Franz Faul, Christian-Albrechts-Universitat, Kiel, Germany) according to Chen et al*.* [17], who demonstrated a difference in inclination of articular tubercle among three classes of vertical skeletal patterns in skeletal Class III malocclusion: hyperdivergent (37.48° ± 1.44°), normodivergent (36.02° ± 6.53°), and hypodivergent (46.65° ± 8.44°). The minimum sample size required to identify a difference among groups using analysis of variance (ANOVA) was 25 images for each subgroup, with a power of 90% and a significance level of 5%.

Under the premise of patients’ informed consent, data from 2076 adolescents’ pre-treatment orthodontic records of CBCT scans between February 2018 and July 2021 were obtained from the Stomatological Hospital of Chongqing Medical University. Inclusion criteria were as follows: (1) 10 ≤ age < 20 years; (2) ANB angle < − 1° for the skeletal class III group; − 1° ≤ ANB angle < 4° and 23° < FH-GoGn < 30° for the control group [19, 20]; (3) symmetrical facial appearance; (4) appropriate alignment with minimal crowding of ≤ 4 mm; and (5) all permanent teeth, except the third molar teeth, had erupted. The exclusion criteria were as follows: (1) history of orthodontic or/and restorative treatment, (2) history of dentofacial trauma or/and surgery in the craniofacial region, (3) transverse discrepancies with functional displacement based on clinical dental records, (4) congenital craniofacial syndrome or anomaly, (5) history of temporomandibular disorder (TMD) [21], and (6) imaging findings of condylar degeneration (e.g. osteoarthritis, erosion, and condylar hyperplasia).

The study included 90 adolescents with skeletal Class III malocclusion (age: 15.04 ± 2.89 years) and 30 controls (age: 14.83 ± 3.05 years). CBCT images were acquired for various clinical reasons, including orthodontic treatment, extraction of impacted teeth, and resolution of the third molars. No further radiologic examinations were performed. The skeletal Class III malocclusion group was further subdivided into a number of subgroups based on sex (male and female), age (early adolescence [10–14 years, 11.63 ± 1.45 years], middle adolescence [14–17 years, 15.37 ± 0.85 years], and late adolescence [17–20 years, 18.13 ± 0.86 years]) [22], and vertical skeletal pattern (hypodivergent group [FH-GoGn ≤ 23°, 18.84° ± 2.48°], normodivergent group [FH-GoGn of 23°–30°, 24.88° ± 1.84°], and hyperdivergent group [FH-GoGn ≥ 30°, 32.34° ± 2.07°]) [20]. The sample distributions of the skeletal Class III malocclusion group and the control group are listed in Table 1 and Additional file 1, respectively.

**Table 1:** Sample distribution in the skeletal Class III malocclusion group

Variables	Sex		Age			Vertical skeletal pattern		
			Early	Middle	Late	Hyperdivergent	Normodiergent	Hypodivergent
	Male	Female	10 to < 14	14 to < 17	17 to < 20	FH-GoGn ≥ 30°	23° < FH-GoGn < 30°	FH-GoGn ≤ 23°
Sex								
Male	43		14	12	17	14	13	16
Female		47	16	18	13	16	17	14
Age								
Early	14	16				10	10	10
Middle	12	18				10	10	10
Late	17	13				10	10	10
Vertical skeletal pattern								
Hyperdivergent	14	16	10	10	10			
Normodivergent	13	17	10	10	10			
Hypodivergent	16	14	10	10	10			
Total (each)	90		30	30	30	30	30	30

### Measurements

The KaVo 3DeXam CT system (KaVo, Biberach, Germany; 120 kV, 5 mA, and field of view of 16 × 17 cm^2^) was used to collect all of the CBCT images. The Frankfort plane was set to be parallel to the floor, and CBCT images were captured while the patients were in maximum dental occlusion. Digital Imaging and Communication in Medicine data were imported and reconstructed using Dolphin 11.9 software (Dolphin Imaging, Chatsworth, CA, USA) for further processing and analysis.

**Fig. 1:**
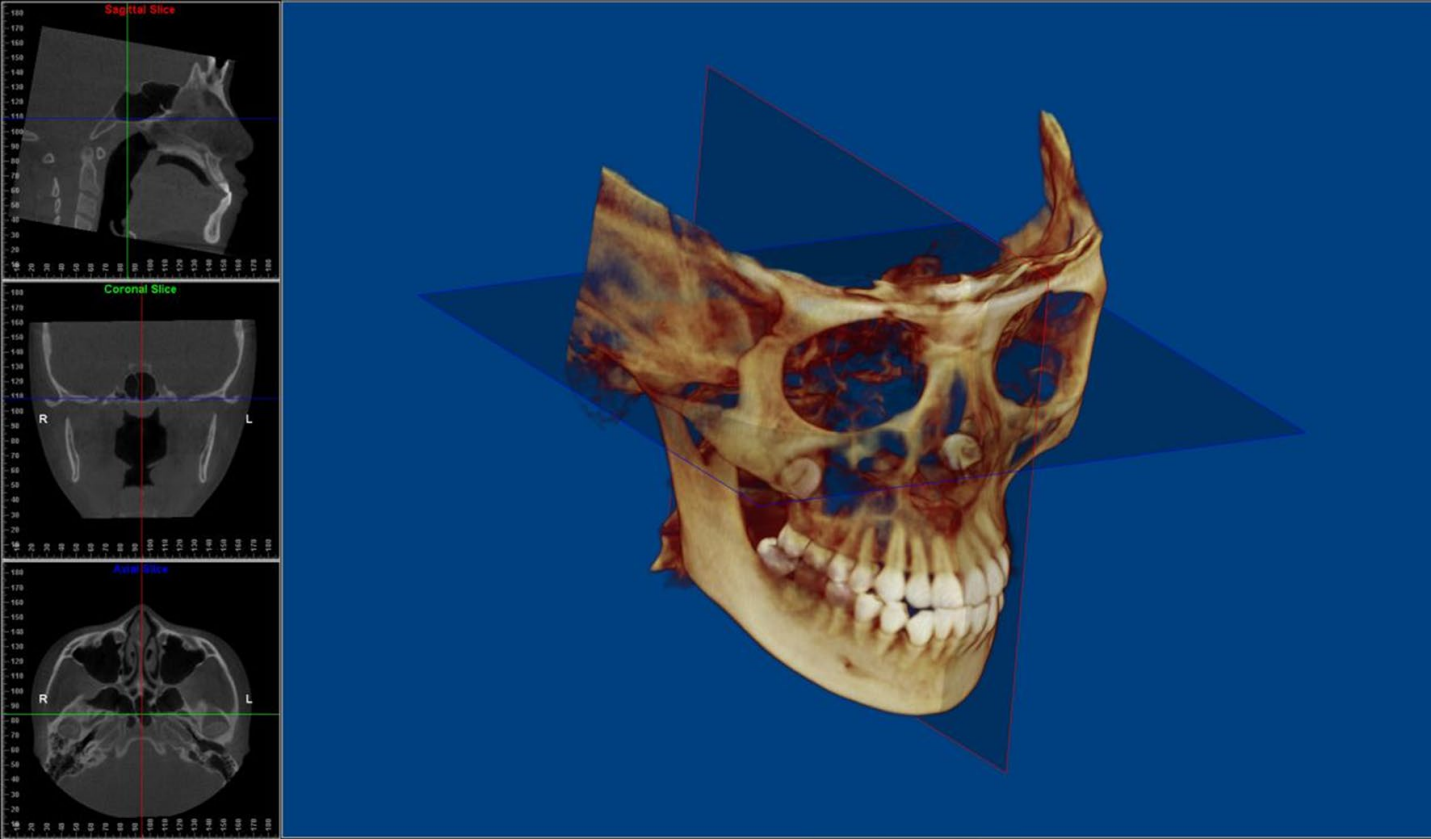
Head orientation on cone-beam computed tomography

**Fig. 2:**
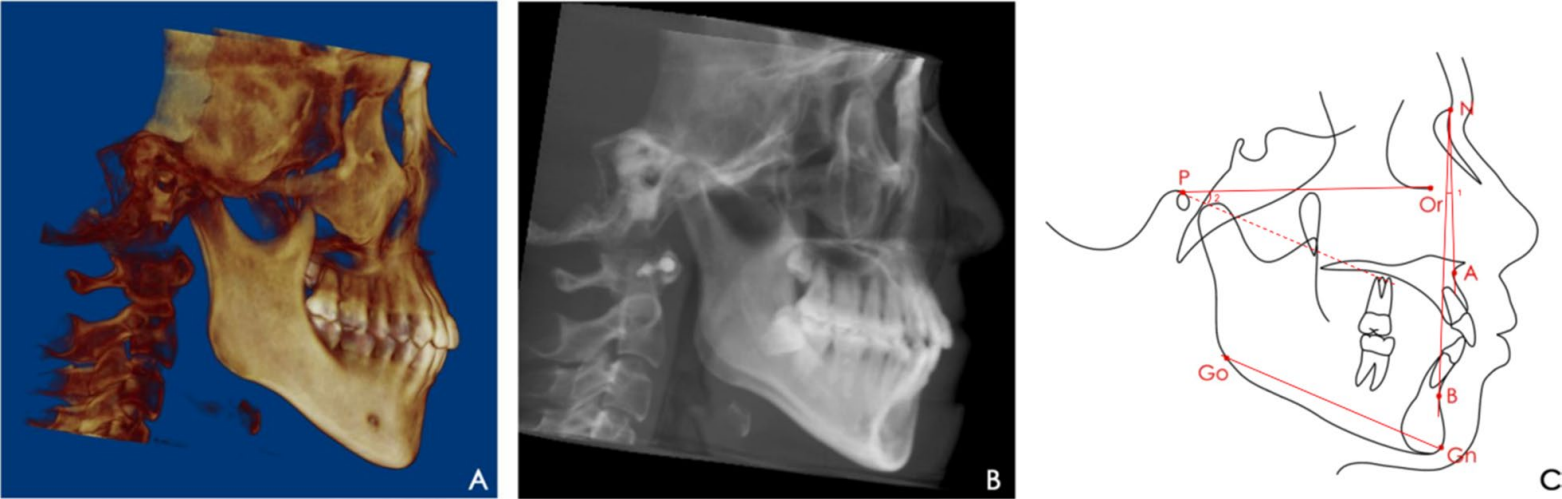
Linear and angular measurements in constructed lateral images. (**a**) head-orientation images; (**b**) constructed lateral images; (**c**) landmarks and measurements in lateral images: A, Point A; B, Point B; N, nasion; Or, orbitale; P, porion; Gn, gnathion; Go, gonion; 1, subspinale-nasion-supramental angle (ANB); 2, Frankfort horizontal plane-gonion-gnathion angle (FH-GoGn)

The image orientation and measurements followed the same procedures as described previously [10, 14, 23]. To standardise head-orientation images, the sagittal plane was adjusted to reflect the midsagittal plane as bisecting symmetrical facial structures. The skull was repositioned using the Frankfort horizontal plane, which was defined by the highest point of the right meatus acusticus externus and the lowest point of the left and right orbital rims (Fig. 1). Constructed lateral images were obtained to evaluate the anteroposterior and vertical skeletal features of each participant radiographically using The Build X-Rays Tool in the Dolphin Imaging program. The landmarks and measurements are shown in Fig. 2.

**Fig. 3:**
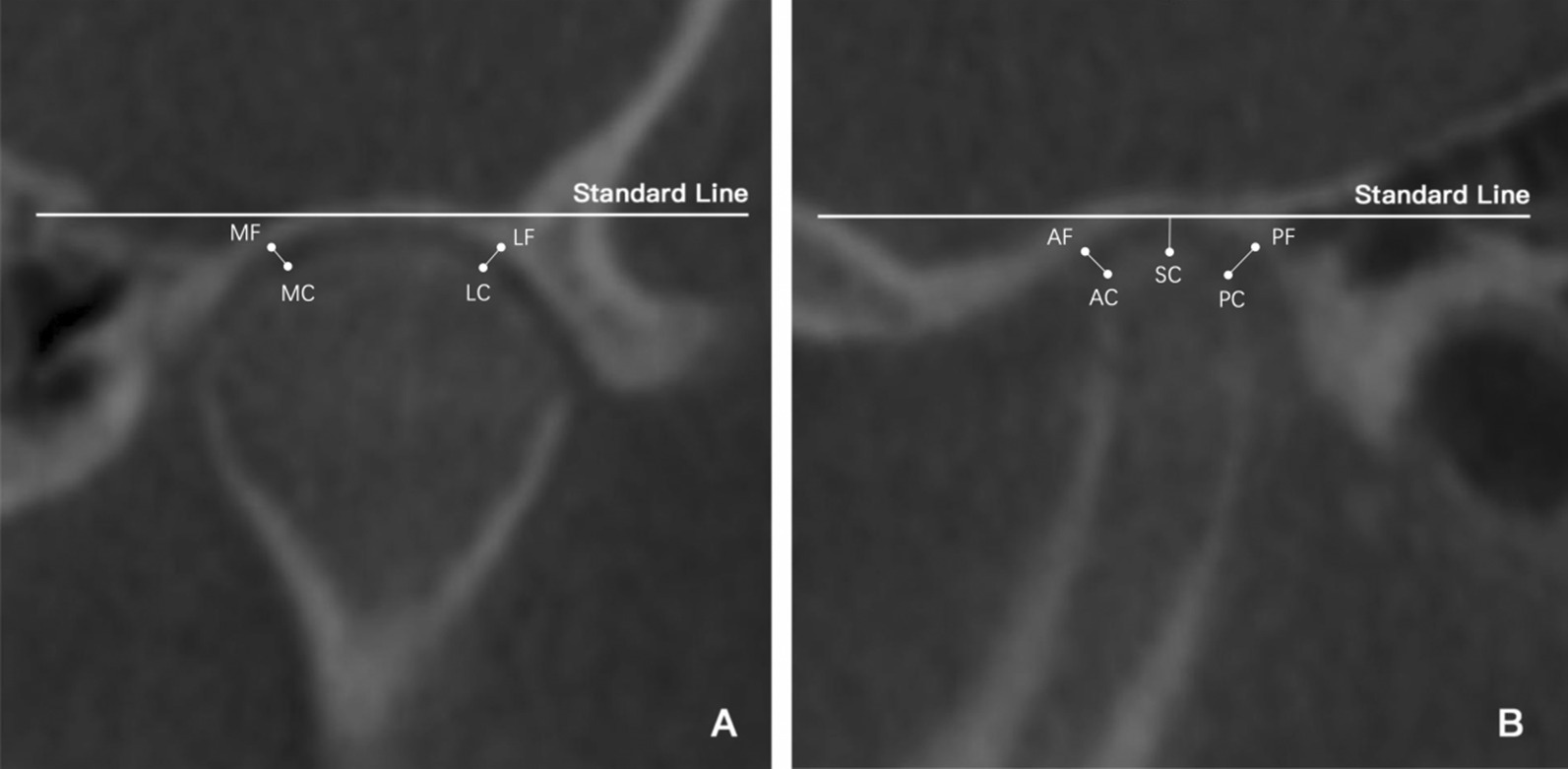
Measurements of TMJ spaces. (**a**) MS, medial space; LS, lateral space; (**b**) SS, superior space; AS, anterior space; PS, posterior space. TMJ, temporomandibular joint

**Fig. 4:**
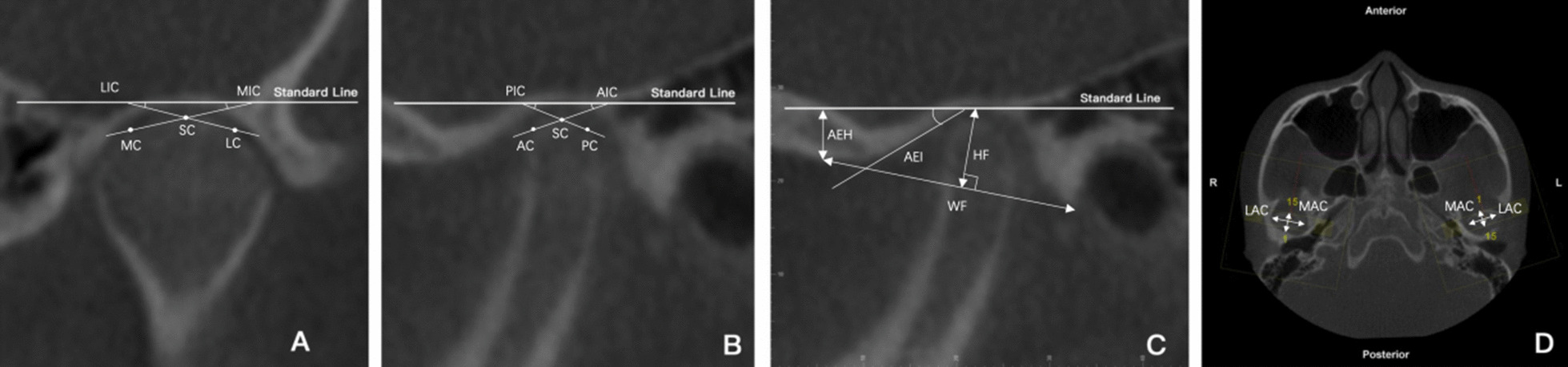
Measurements of TMJ shape. (**a**) MIC, medial inclination of the condyle; LIC, lateral inclination of the condyle; (**b**) AIC, anterior inclination of the condyle; PIC, posterior inclination of the condyle; (**c**) WF, width of the fossa; HF, height of the fossa; AEH, articular eminence height; AEI, articular eminence inclination; (**d**) LAC, long axis of the condyle; MAC, minor axis of the condyle. TMJ, temporomandibular joint

**Fig. 5:**
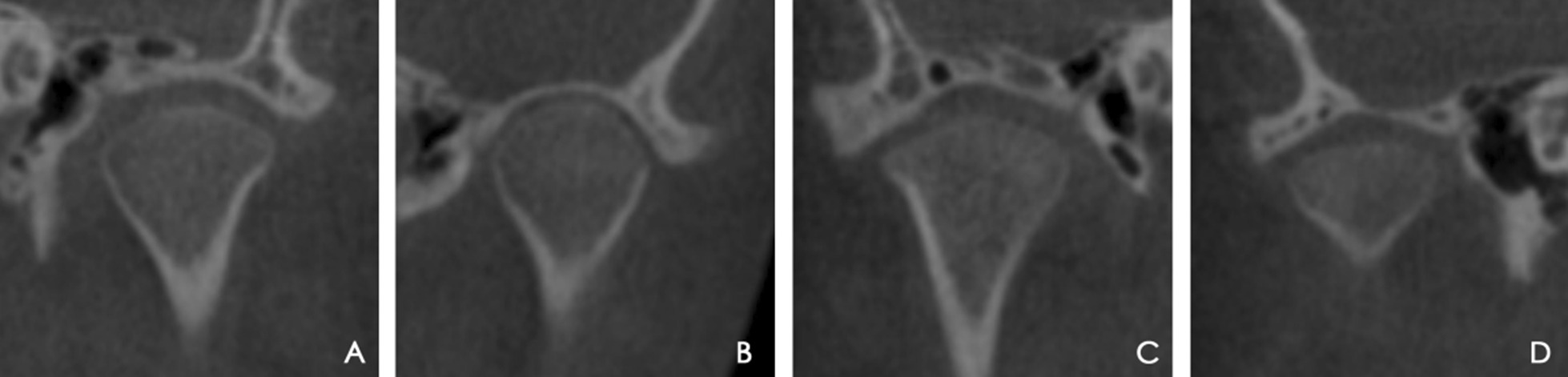
Shape of the condyle on the coronal plane: convex (**a**), round (**b**), angled (**c**), and flat (**d**)

For TMJ evaluation, the thickness of the slices was set to 0.5 mm. The Standard Line was defined as a line tangent to the highest point of the mandibular fossa and parallel to the Frankfort horizontal plane. The liner and angular measurements were assessed from the axial, coronal, and sagittal slices with the largest condylar diameters (Figs. 3, 4): TMJ space (TMJS) (medial space [MS], lateral space [LS], anterior space [AS], posterior space [PS], and superior space [SS]), inclination of the mandibular condyle (the medial inclination of the mandibular condyle [MIC], lateral inclination of the mandibular condyle [LIC], anterior inclination of the mandibular condyle [AIC], posterior inclination of the mandibular condyle [PIC]), width and depth of the glenoid fossa (WF, and height of the glenoid fossa [HF]), height and inclination of the articular eminence (articular eminence height [AEH] and articular eminence inclination [AEI]), and diameter of the condylar head (long axis of the condylar head [LAC] and minor axis of the condylar head [MAC]). TMJS was measured as the shortest distance between two points: MS, medial fossa (MF) and medial condyle (MC); LS, lateral fossa (LF) and lateral condyle (LC); AS, anterior fossa (AF) and anterior condyle (AC); PS, posterior fossa (PF) and posterior condyle (PC). SS was the distance between the superior condyle (SC) and the standard line. MIC was the angle formed by the standard line and the line that connected the MC and SC; LIC was the angle formed by the standard line and the line that connected the LC and SC; AIC was the angle formed by the standard line and the line that connected the AC and SC; PIC was the angle formed by the standard line and the line that connected the LC and SC. HF was the vertical distance between the highest point of the glenoid fossa and the line connecting the lowest point of the articular tubercle to the lowest point of the postglenoid process. WF was the distance between the glenoid tubercle and the postglenoid process at their lowest points. AEH is the distance perpendicular to the standard line through the glenoid eminence's lowest point. AEI was the angle formed by the standard line and the tangent line on the posterior glenoid eminence surface. LAC was the largest mediolateral diameter of the condyle; MAC was the largest anteroposterior diameter of the condyle. The shapes of the mandibular condyle in the coronal plane were classified into four types (i.e. round, convex, flat, or angled) according to Yale et al*.* [24] (Fig. 5).

A total of 240 TMJs were measured and analysed, with each side of the TMJ being evaluated separately. The formula PS-AS/PS + AS/100, which was developed by Pullinger and Hollender [25], was able to provide an accurate assessment of the anteroposterior condylar position on the sagittal plane. The mandibular condyle was found to be anteriorly positioned when the value was positive and posteriorly positioned when the value was negative.

### Statistical analyses

All measurements were taken by a single investigator with experience in evaluating TMJ regions. To determine intra-examiner reliability, each case was re-examined twice within three weeks. Intra-class correlation coefficients of 0.97–1.00 were obtained, indicating that the measurements were reproducible.

The mean and standard deviation of each variable was analysed and presented as descriptive statistics. The Kolmogorov–Smirnov test showed that all data followed a normal distribution. For intergroup analyses, we compared the joint measurements between the controls and skeletal Class III malocclusion with different vertical skeletal patterns using an independent sample *t*-test. For intra-group analyses, an independent sample *t*-test, an ANOVA, and a paired *t*-test were performed to compare the features of the mandibular condyle and articular fossa in participants with skeletal Class III malocclusion based on sex, age, side, and vertical skeletal patterns. Pearson correlation analysis was used to determine the correlation between the ANB or FH-GoGn of adolescents with skeletal Class III malocclusion and measurement items relating to condyle-fossa relationships. Percentages of shapes were assessed according to different vertical skeletal patterns. IBM SPSS Statistics software was used to analyse all data (ver. 26.0, IBM Corp, Armonk, NY, USA). A *P* value of < 0.05 was considered significant.

This study was conducted and reported in accordance with the STROBE criteria for Observational Studies (Additional file 2).

## Results

For intergroup analyses, the measurements in skeletal Class III malocclusion with different vertical skeletal patterns compared with Class I skeletal normodivergent patterns (control group) are presented in Table 2. Regarding the condylar position, no substantial differences were found between the control and skeletal Class III normodivergent groups, and between the control and skeletal Class III hypodivergent patterns. However, statistically significant differences were found in the MS (*P* = 0.032), SS (*P* = 0.000), AS (*P* = 0.003), and PS (*P* = 0.019) between Class I normodivergent and Class III hyperdivergent skeletal patterns. With respect to the condylar and glenoid fossa morphology, significant differences were observed in the MIC (*P* = 0.046) between the control and skeletal Class III hyperdivergent groups. In addition, the AIC (*P* = 0.003; *P* = 0.001) and AEI (*P* = 0.012;* P* = 0.001) were considerably flatter in individuals with skeletal Class III normodivergent and hyperdivergent patterns than in controls. While the HF (*P* = 0.004; *P* = 0.001) and AEH (*P* = 0.039; *P* = 0.011) were higher in the control group than in the skeletal Class III normodivergent and hyperdivergent group, the WF (*P* = 0.000) was greater in all skeletal Class III subgroups than in the control group.

**Table 2:** Comparison between controls and skeletal Class III malocclusion with different vertical skeletal patterns

Measurements	Control group		Hypodivergent group		Normodivergent group		Hyperdivergent group		Independent sample *t*-test (*P* value)		
	Mean	SD	Mean	SD	Mean	SD	Mean	SD	Control-Hypodivergent	Control-Normodivergent	Control-Hyperdivergent
Skeletal measurement											
ANB	2.50	1.02	− 3.88	2.45	− 3.40	1.93	− 2.28	1.81	0.000***	0.000***	0.000***
FH-GoGn	25.93	2.78	18.84	2.48	24.88	1.84	32.34	2.07	0.000***	0.090	0.000***
TMJ space											
MS	2.27	0.52	2.24	0.59	2.09	0.45	1.94	0.63	0.808	0.157	0.032*
LS	1.98	0.42	2.00	0.58	1.80	0.38	1.78	0.47	0.899	0.076	0.079
SS	2.57	0.66	2.81	0.73	2.39	0.59	1.87	0.63	0.190	0.253	0.000***
AS	2.11	0.62	2.02	0.77	1.89	0.46	1.70	0.39	0.614	0.124	0.003**
PS	2.16	0.52	1.94	0.51	1.95	0.41	1.85	0.45	0.106	0.094	0.019*
Anteroposterior condylar position	0.02	0.16	0.00	0.19	0.02	0.13	0.04	0.12	0.643	0.956	0.521
TMJ shape											
MIC	17.75	3.17	17.72	4.13	16.46	3.73	15.80	4.16	0.976	0.156	0.046*
LIC	18.09	4.83	18.10	4.98	16.71	4.17	16.15	5.01	0.994	0.243	0.133
AIC	31.27	6.68	28.67	5.89	26.52	5.05	25.68	5.70	0.115	0.003**	0.001**
PIC	27.11	5.67	25.84	5.50	28.30	5.34	28.68	5.90	0.379	0.408	0.299
HF	7.02	1.12	7.04	1.20	6.21	0.98	6.15	0.73	0.965	0.004**	0.001**
WF	15.78	3.05	19.35	1.80	18.69	1.57	18.40	2.12	0.000***	0.000***	0.000***
AEH	6.16	1.29	6.63	1.48	5.50	1.11	5.43	0.77	0.192	0.039*	0.011*
AEI	50.97	11.3	55.72	12.86	44.27	8.54	40.43	11.42	0.134	0.012*	0.001**
LAC	18.62	2.43	18.71	1.66	18.65	1.92	17.65	2.05	0.872	0.963	0.099
MAC	9.28	1.02	8.93	0.93	8.94	0.86	8.58	0.87	1.000	0.167	0.167

*P*, *P* value; MS, medial space; LS, lateral space; SS, superior space; AS, anterior space; PS, posterior space; MIC, medial inclination of the mandibular condyle; LIC, lateral inclination of the mandibular condyle; AIC, anterior inclination of the mandibular condyle; PIC, posterior inclination of the mandibular condyle; WF, width of the glenoid fossa; HF, height of the glenoid fossa; AEH, articular eminence height; AEI, articular eminence inclination; LAC, long axis of the condyle; MAC, minor axis of the condyle

*P < 0.05; **P < 0.01; ***P < 0.001

Table 3 shows the averages, standard deviations, and comparisons of linear and angular measurements based on sex, age, and side in skeletal Class III malocclusion. Statistically significant differences were found in SS, AEI, and LAC according to the sex of adolescents with skeletal Class III malocclusion. The SS, AEI, and LAC were considerably larger in males than in females (*P* = 0.002, *P* = 0.011, and *P* = 0.002, respectively). ANOVA revealed a substantial difference in PS (*P* = 0.001) and the anteroposterior condylar position (*P* = 0.005) according to age. This finding indicates that the condylar position was more posterior in late adolescence. Significant differences were also identified in the HF (*P* = 0.010), WF (*P* = 0.002), AEH (*P* = 0.027), and AEI (*P* = 0.039) among different age groups. No statistical differences were observed in TMJ positional and dimensional measurements between the left and right sides.

**Table 3:** Measurements of TMJ according to sex, age, and side in skeletal Class III malocclusion

	Total			Males		Females		*P*	Early		Middle		Late		*P*	Right		Left		*P*
	(n = 90)	Ratio	SD	(n = 43)	SD	(n = 47)	SD		(n = 30)	SD	(n = 30)	SD	(n = 30)	SD		(n = 90)	SD	(n = 90)	SD	
TMJ space																				
MS	2.09	1.00	0.57	2.19	0.62	1.99	0.51	0.098	2.21	0.62	2.17	0.53	1.89	0.53	0.065	2.08	0.66	2.10	0.84	0.806
LS	1.86	0.89	0.49	1.92	0.55	1.80	0.43	0.268	1.91	0.49	1.84	0.43	1.82	0.55	0.744	1.76	0.55	1.95	0.74	0.052
SS	2.36	1.25	0.75	2.61	0.79	2.12	0.64	0.002**	2.22	0.72	2.30	0.81	2.55	0.71	0.211	2.32	0.80	2.40	1.22	0.604
AS	1.88	1.00	0.57	1.92	0.64	1.82	0.50	0.379	1.79	0.37	1.88	0.55	1.93	0.75	0.640	1.84	0.63	1.90	0.82	0.596
PS	1.91	1.01	0.45	1.98	0.50	1.86	0.40	0.216	2.15	0.46	1.86	0.38	1.74	0.43	0.001**	1.87	0.52	1.96	0.70	0.327
Anteroposterior condylar position	0.02		0.15	0.02	0.16	0.02	0.15	0.915	0.88	0.11	0.00	0.13	− 0.03	0.19	0.005**	0.01	0.19	0.03	0.26	0.594
TMJ shape																				
MIC	16.66		4.05	16.72	3.99	16.61	4.14	0.894	16.65	4.00	17.44	3.18	15.90	4.79	0.341	15.92	5.71	17.40	7.80	0.150
LIC	16.99		4.76	16.54	4.98	17.39	4.55	0.402	18.07	4.51	17.58	3.64	15.31	5.60	0.055	16.01	4.78	17.96	8.29	0.056
AIC	26.96		5.64	26.63	5.74	27.26	5.58	0.599	25.09	5.65	27.51	6.09	28.28	4.78	0.07	26.58	7.85	27.34	10.19	0.574
PIC	27.60		5.67	28.23	5.61	27.03	5.72	0.318	26.02	3.59	28.05	4.94	28.75	7.56	0.154	26.74	7.90	28.47	8.68	0.163
HF	6.47		1.06	6.60	1.25	6.34	0.84	0.244	6.00	0.88	6.76	0.84	6.64	1.27	0.010*	6.6	1.19	6.33	1.44	0.175
WF	18.81		1.87	18.68	2.04	18.94	1.71	0.510	17.91	1.94	19.00	1.76	19.53	1.56	0.002**	18.65	1.80	18.98	3.40	0.425
AEH	5.85		1.27	5.89	1.47	5.82	1.07	0.804	5.35	1.16	6.09	1.14	6.13	1.39	0.027*	5.68	1.32	6.03	2.29	0.21
AEI	46.81		12.76	50.34	13.65	43.58	11.07	0.011*	43.03	12.87	46.14	9.17	51.26	14.63	0.039*	44.86	13.11	48.76	21.22	0.14
LAC	18.33		1.93	18.97	1.91	17.75	1.77	0.002**	18.09	1.64	18.16	2.14	18.75	1.97	0.358	18.56	2.13	18.11	3.49	0.406
MAC	8.82		0.89	8.92	1.03	8.72	0.74	0.287	8.71	0.81	8.96	0.98	8.79	0.89	0.556	8.86	0.95	9.00	1.50	0.053

*P*, *P* value; MS, medial space; LS, lateral space; SS, superior space; AS, anterior space; PS, posterior space; MIC, medial inclination of the mandibular condyle; LIC, lateral inclination of the mandibular condyle; AIC, anterior inclination of the mandibular condyle; PIC, posterior inclination of the mandibular condyle; WF, width of the glenoid fossa; HF, height of the glenoid fossa; AEH, articular eminence height; AEI, articular eminence inclination; LAC, long axis of the condyle; MAC, minor axis of the condyle

^*^P < 0.05; **P < 0.01; ***P < 0.001

Significant differences in the SS were identified among three different skeletal vertical patterns in adolescents with skeletal Class III malocclusion (*P* = 0.000) (Table 4). The hypodivergent group had more inferior condyles than the normodivergent (*P* = 0.000) and hyperdivergent (*P* = 0.037) groups. When the TMJ shape of these groups was compared, there were statistically substantial differences in HF (*P* = 0.001), AEH (*P* = 0.000), and AEI (*P* = 0.000). The HF was dramatically greater in the brachycephalic profiles than in the mesocephalic (*P* = 0.005) and dolichocephalic (*P* = 0.002) profiles. The AEH and AEI were significantly larger in the brachycephalic profiles than in the mesocephalic (*P* = 0.000) and dolichocephalic (*P* = 0.000) profiles.

**Table 4:** Mean values of TMJ measurements according to vertical skeletal patterns in participants with skeletal Class III malocclusion

Measurements	Hypodivergent		Normodivergent		Hyperdivergent		ANOVA	Multiple comparison Tukey's HSD test (*P* value)		
	Mean	SD	Mean	SD	Mean	SD		Hypodivergent–Normodivergent	Hypodivergent–Hyperdivergent	Normdivergent–Hyperdivergent
Skeletal measurement										
FH-GoGn	18.84	2.48	24.88	1.84	32.34	2.07	0.000***	0.000***	0.000***	0.000***
TMJ space										
MS	2.24	0.59	2.09	0.45	1.94	0.63	0.138	0.582	0.115	0.568
LS	2.00	0.58	1.80	0.38	1.78	0.47	0.149	0.235	0.185	0.990
SS	2.81	0.73	2.39	0.59	1.87	0.63	0.000***	0.037*	0.000***	0.009**
AS	2.02	0.77	1.89	0.46	1.70	0.39	0.098	0.662	0.082	0.398
PS	1.94	0.51	1.95	0.41	1.85	0.45	0.676	0.993	0.761	0.693
Anteroposterior condylar position	0.00	0.19	0.02	0.13	0.04	0.12	0.516	0.822	0.483	0.843
TMJ shape										
MIC	17.72	4.13	16.46	3.73	15.80	4.16	0.177	0.449	0.160	0.801
LIC	18.10	4.98	16.71	4.17	16.15	5.01	0.267	0.497	0.255	0.890
AIC	28.67	5.89	26.52	5.05	25.68	5.70	0.105	0.295	0.099	0.829
PIC	25.84	5.50	28.30	5.34	28.68	5.90	0.107	0.208	0.125	0.963
HF	7.04	1.20	6.21	0.98	6.15	0.73	0.001**	0.005**	0.002**	0.970
WF	19.35	1.80	18.69	1.57	18.40	2.12	0.13	0.351	0.12	0.818
AEH	6.63	1.48	5.50	1.11	5.43	0.77	0.000***	0.001**	0.000***	0.973
AEI	55.72	12.86	44.27	8.54	40.43	11.42	0.000***	0.000***	0.000***	0.377
LAC	18.71	1.66	18.65	1.92	17.65	2.05	0.055	0.992	0.080	0.105
MAC	8.93	0.93	8.94	0.86	8.58	0.87	0.217	0.329	0.343	1.000

*P*, *P* value; MS, medial space; LS, lateral space; SS, superior space; AS, anterior space; PS, posterior space; MIC, medial inclination of the mandibular condyle; LIC, lateral inclination of the mandibular condyle; AIC, anterior inclination of the mandibular condyle; PIC, posterior inclination of the mandibular condyle; WF, width of the glenoid fossa; HF, height of the glenoid fossa; AEH, articular eminence height; AEI, articular eminence inclination; LAC, long axis of the condyle; MAC, minor axis of the condyle

*P < 0.05; **P < 0.01; ***P < 0.001

As shown in Table 5, ANB had a negative correlation with LIC (*P* = 0.019) and SS (*P* = 0.036) in participants with skeletal Class III. FH-GoGn was negatively correlated with SS (*P* = 0.000), AS (*P* = 0.006), MS (*P* = 0.016), MIC (*P* = 0.048), HF (*P* = 0.001), AEH (*P* = 0.000), AEI (*P* = 0.000), and LAC (*P* = 0.032).

**Table 5:** Correlation analysis of ANB and FH-GoGn

Variables	ANB (n = 90)		FH-GoGN (n = 90)	
	r	*P*	r	*P*
TMJ space				
MS	− 0.098	0.360	− 0.252	0.016*
LS	− 0.033	0.755	− 0.199	0.061
SS	− 0.221	0.036*	− 0.537	0.000***
AS	− 0.075	0.480	− 0.296	0.005**
PS	− 0.088	0.411	− 0.134	0.207
Anteroposterior condylar position	0.001	0.990	0.128	0.231
TMJ shape				
MIC	− 0.106	0.321	− 0.209	0.048*
LIC	− 0.246	0.019*	− 0.163	0.125
AIC	− 0.190	0.730	− 0.187	0.077
PIC	0.093	0.382	0.205	0.052
HF	− 0.115	0.282	− 0.338	0.001**
WF	− 0.171	0.106	− 0.145	0.172
AEH	− 0.020	0.848	− 0.397	0.000***
AEI	− 0.202	0.057	− 0.563	0.000***
LAC	− 0.162	0.127	− 0.235	0.026*
MAC	0.040	0.710	− 0.110	0.304

R, R statistics; *P*, *P* value; MS, medial space; LS, lateral space; SS, superior space; AS, anterior space; PS, posterior space; MIC, medial inclination of the mandibular condyle; LIC, lateral inclination of the mandibular condyle; AIC, anterior inclination of the mandibular condyle; PIC, posterior inclination of the mandibular condyle; WF, width of the glenoid fossa; HF, the height of the glenoid fossa; AEH, articular eminence height; AEI, articular eminence inclination; LAC, long axis of the condyle; MAC, minor axis of the condyle

*P < 0.05; **P < 0.01; ***P < 0.001

Table 6 shows the subjective assessment of the fossa and condylar form. The convex shape was the most common in all groups on coronal images, followed by round, angled, and flat shapes.

**Table 6:** Percentage of condylar shapes on coronal images

Shape	Control group	Class III	Class III	Class III
		Hypodivergent	Normodivergent	Hyperdivergent
	No. (%)	No. (%)	No. (%)	No. (%)
Convex	53 (88.3)	38 (63.3)	37 (61.7)	41 (68.3)
Round	3 (5)	16 (26.7)	20 (33.3)	14 (23.3)
Angled	2 (3.3)	4 (6.7)	2 (3.3)	3 (5)
Flat	2 (3.3)	2 (3.3)	1 (1.7)	2 (3.3)

## Discussion

Long-term effects of skeletal Class III malocclusion on the TMJ, jaw movement, and masticatory system may result from an abnormal sagittal relationship between the maxilla and mandible. Because the TMJ is subject to the tension or compression forces by the tissues that surround it, the condyle-fossa relationship may be compromised, thereby allowing for continuous adaptability to functional changes in surrounding tissues via remodelling processes [8, 26]. Indeed, changes in morphological structures and spatial relationships of the TMJ, as well as the links to existing skeletal malocclusions, have been demonstrated in many studies [6–17]. In adolescents with skeletal Class III malocclusion, evaluating TMJ characteristics may help dental professionals detect radiographic abnormalities, allowing for better treatment planning. Consequently, accurate measurements of these imaging values in conjunction with clinical examinations may be highly essential for the diagnosis and treatment of skeletal Class III malocclusion [27].

In recent years, CBCT has been widely used in the diagnostic assessment of a variety of TMJ conditions. Some researchers have demonstrated the superiority of this imaging modality and accuracy over conventional radiographic examinations in the assessment of the TMJ region [28]. Hence, CBCT assessments of the location and structures of the TMJ are accurate and reliable.

Intergroup comparisons showed non-substantial differences in the position of condyle between the control (skeletal Class I normodivergent patterns) and skeletal Class III normodivergent groups, and between the control and hypodivergent patterns. However, there were significant changes in the TMJ position in skeletal Class III hyperdivergent cases, where the skeletal Class III hyperdivergent group had a smaller distance between the glenoid fossa and mandibular condyle compared with the controls; this may indicate that significant position variations of the condyle-fossa relationships can be found in skeletal Class III adolescents with a high-angle vertical pattern. Several factors determine the position of the condyle in the fossa, including disc thickness and the surrounding tissues. Katsavrias et al*.* [8], Seren et al*.* [9], and Cohlmia et al*.* [11] found that the condyle was positioned more anteriorly and superiorly in Class III skeletal patterns, while Paknahad et al*.* [7] demonstrated that the condyle-fossa spatial relationship was not statistically significant in Class III patients compared with Class I counterparts. The relationships between craniofacial morphology and condylar position have long been debated. Some studies, including our present study, showed a significant association between the distribution of condylar position and facial morphology [6, 8, 9]. However, numerous other studies found no correlation between condylar position and skeletal patterns, implying that the condylar position is independent of the skeletal pattern [7, 10]. The study design, research approach, and imaging technique could account for these contradictory findings. Notably, the condylar position can be evaluated by examining the space between the condyle and fossa on radiographic images. In our study, the mean ratio of AS to SS to PS was 1.00 to 1.25 to 1.01 in adolescents with skeletal Class III malocclusion, suggesting that the condylar position in Class III malocclusion is basically concentric. Generally speaking, concentric condyle positioning is considered ideal, but this finding is still debatable [12, 29]. Thus, the TMJS in skeletal Class III malocclusion should be further investigated.

Regarding fossa and condyle morphology, the current study found that the fossa was wider and shallower in individuals with skeletal Class III malocclusion with mesocephalic and dolichocephalic profiles than in controls; this finding is compatible with that in the investigation by Katsavrias et al*.* [8]. Moreover, in line with previous findings [8, 30], the inclination of the articular eminence and the anterior inclination of the condyle were flatter in participants with Class III skeletal normodivergent and hyperdivergent patterns than in those with Class I skeletal normodivergent patterns. Different stress magnitudes, directions, and distributions on the condyle could explain the variations in condyle morphology, and different maxillofacial morphologies may influence the forces pressing on the TMJ. For example, Ueki et al*.* [31] and Tanne et al*.* [32] demonstrated that different sagittal and vertical skeletal patterns induced different functional loads imposed on the TMJ, which are capable of modifying the TMJ’s anatomical features. These findings suggest that sagittal and vertical discrepancies may have an impact on the morphological features of the TMJ.

In the present study, intra-group comparisons revealed that females’ condyles were closer to the articular fossa in the vertical direction than males’ condyles in skeletal Class III malocclusion, demonstrating that there is a significant difference in the superior joint space between the sexes; the findings are consistent with those in previous studies [12, 13]. However, Chae et al*.* [10] reported no statistically substantial difference between the sexes in terms of joint space. Additionally, sex dimorphism in the TMJ structures was observed in the current study, and males with skeletal Class III malocclusion had a significantly larger inclination of the articular eminence and LAC than females. Consistently, Chae et al*.* [10] found that males had a larger vertical height of the articular tubercle than females. It has been proposed that male hormones increase muscle strength, which may be transmitted to the TMJ complex, thereby resulting in a higher level of bone remodelling in TMJ morphology [33]. This remodelling could explain the greater inclination of the eminence and the condylar head complex in males. Moreover, there were no noticeable differences in TMJ configurations between the right and left sides, implying that the positional and morphological features of bilateral condyles are essentially symmetrical in skeletal Class III malocclusion, as described by Rodrigues et al*.* [26]. In contrast, Katsavrias et al*.* [8] found that the superior joint space was larger on the right side than on the left side in the Class III malocclusion sample. This finding may be related to the difference in ethnicity or probably due to different measurement methods.

Regarding joint spaces, the PS was smaller in late adolescence than in early and middle adolescence. Moreover, a statistically substantial difference in anteroposterior condylar position was found among three different age groups. These findings suggest that the condyle is located more posteriorly in the late period of adolescence, and the distribution on the mandibular condyle may be influenced by age, as described by Cohlmia et al. [11]. Regarding the HF and WF, the articular fossa of older participants was wider and shallower than that of younger participants. This change could be attributed to growth and development, as demonstrated by Li et al*.* [14]. Additionally, in our study, the AEH and AEI revealed substantial differences among different age groups, suggesting that the AEH and AEI exhibit a sustained increase throughout adolescence; these results are consistent with previous findings [34, 35]. The posterior attachment and innervated tissues may be compressed by the backward location of the condyle [36], and a steeper articular tubercle may be linked to articular disk dislocation [34, 37]; these factors can affect TMJ function. The higher frequency of the backward condylar position and excessively inclined eminence suggests that the anatomical abnormalities of TMJ structures are more prevalent in late adolescence, which is consistent with the increased prevalence of TMD in this period [38]. This assessment can be clinically valuable in terms of susceptibility to TMD.

In this study, we found no statistically substantial differences in the anterior or posterior condylar position among different vertical skeletal patterns in adolescents with skeletal Class III malocclusion but a weak relationship between the vertical pattern and AS of the TMJ. This may indicate that the anteroposterior position of the condyle in the fossa may not influence the facial morphology in skeletal Class III malocclusion. Consistently, several studies [10, 16, 39] demonstrated that the sagittal position of the condyle did not differ significantly among vertical skeletal patterns. Nevertheless, Paknahad et al*.* [15] showed the anterior position of the condyle in individuals with a long face. Differences in measuring methods, age ranges, and sample selections for participants may also be important factors contributing to the controversy. Additionally, the condyle was placed more inferiorly in brachycephalic profiles than in dolichocephalic profiles in adolescents with skeletal Class III malocclusion, which is similar to the findings in previous studies [6, 8, 16]. Furthermore, our findings revealed a significant correlation between SS and vertical skeletal patterns. Burke et al*.* [39] noticed that the more superior position of the condyle in dolichocephalic profiles reflected a reduction in the condylar tissue, which can result in decreased condylar growth potential, thereby leading to increased anterior facial height during growth and development. This finding could be used in the future to make predictions regarding the growth patterns of the mandible and its potential for growth.

The morphological features of the TMJ in skeletal Class III malocclusion is affected by vertical craniofacial morphology. In our study, the fossa height was significantly larger in the brachycephalic profiles than in the dolichocephalic profiles. In addition, there were statistically significant interactions between the vertical cephalometric patterns and fossa height; this finding is consistent with that of the study by Park et al*.* [16]. However, Noh et al*.* [40] failed to find these results, probably due to different inclusion criteria. Furthermore, the current research found substantial differences in AEI and AEH among different vertical facial patterns, which is in line with the observations of Costa et al*.* [41], who concluded that the AEH and AEI of individuals with a long face were significantly smaller than those of individuals with a short face. Moreover, we found that vertical skeletal patterns were negatively correlated with AEH and AEI. It is commonly recognised that TMJ structure is determined by the forces acting on it, and TMJ loading varies between individuals with distinct dentofacial morphologies. Meanwhile, the articular eminence is associated with the TMJ’s dynamic function, which is highly dependent on masticatory loads. Notably, steeper eminence inclination indicates increased muscular forces acting on the condyle-fossa complex, resulting in TMJ remodelling [42]. Compared with individuals with a high-angle vertical pattern, individuals with a short face have greater bite forces generated by the contractility of the masticatory muscles during chewing movements, resulting in a difference in the anterior condylar slope and AEI. This finding may explain why the condyle-fossa relationship varies between individuals with various vertical craniofacial profiles. Thus, these findings confirm that in individuals with a low-angle vertical pattern, facial structures and bite forces may impact TMJ configuration. Additionally, the most common shape of condylar morphology on coronal images among different groups was found to be convex in the current study; this result is similar to that in a previous study [43]. These findings indicate that skeletal patterns have no influence on the shape of the condyle.

In addition to its strengths, this study had a few drawbacks. Although the sample size was predetermined in our study, additional investigations involving a greater number of participants are required to validate the current findings. To overcome study design limitations, a more precise TMJ analysis, including measurements of mandibular condylar motion, occlusal force, and stress direction at the TMJ, should be performed.

## Conclusions

Shallower glenoid fossa depth, wider articular fossa width, and flatter mandibular eminence inclination and anterior inclination of the condyle were found in the skeletal Class III normodivergent and hyperdivergent group than in the control group (skeletal class I normodivergent group). In skeletal Class III malocclusion, the condyle was positioned more inferiorly, and the inclination of the mandibular eminence was steeper in males than in females. Individuals in early adolescence exhibited narrower and shallower fossae with flatter articular eminence than those in middle and late adolescence, and the condylar location was more posterior in late adolescence. There were no differences in TMJ components between the left and right sides. The condyle position was inferior, and the articular eminence slope was steeper in brachycephalic profiles than in mesocephalic and dolichocephalic profiles.

In conclusion, there were significant differences in the condyle-fossa relationships between different ages, sexes, and vertical skeletal patterns, but not between the left and right sides of the TMJ, in adolescents with skeletal Class III malocclusion. This finding can be used clinically and radiographically to assess the condyle and glenoid fossa comprehensive features in adolescents with skeletal Class III malocclusion, hence providing a basis for better TMD diagnosis and orthodontic treatment.

## Supplementary Information

**Additional file 1.** Distribution of the control group.

**Additional file 2.** STROBE Statement—checklist of items that should be included in reports of observational studies.

## Data Availability

The data that support the findings of this study are available from the Stomatological Hospital of Chongqing Medical University, but restrictions apply to the availability of these data, which were used under license for the current study, and so are not publicly available. Data are however available from the corresponding author upon reasonable request and with permission of the Stomatological Hospital of Chongqing Medical University.

## Abbreviations

AC: Anterior condyle
AEH: Articular eminence height
AEI: Articular eminence inclination
AF: Anterior fossa
AIC: Anterior inclination of the mandibular condyle
ANOVA: Analysis of variance
AS: Anterior space
CBCT: Cone-beam computed tomography
HF: Height of the glenoid fossa
LAC: Long axis of the condyle
LC: Lateral condyle
LF: Lateral fossa
LIC: Lateral inclination of the mandibular condyle
LS: Lateral space
MAC: Minor axis of the condyle
MC: Medial condyle
MF: Medial fossa
MIC: Medial inclination of the mandibular condyle
MS: Medial space
PC: Posterior condyle
PF: Posterior fossa
PIC: Posterior inclination of the mandibular condyle
PS: Posterior space
SC: Superior condyle
SS: Superior space
TMJ: Temporomandibular joint
TMD: Temporomandibular disorder
TMJS: TMJ space
WF: Width of the glenoid fossa
