# Supplementary material for: Comprehensive positional and morphological assessments of the temporomandibular joint in adolescents with skeletal Class III malocclusion: a retrospective CBCT study
Source: BMC Oral Health. 2023 Feb 7;23:78. doi: 10.1186/s12903-023-02788-4 (PMC9903422; doi:10.1186/s12903-023-02788-4)
Supplement: Supplementary file 1 — Additional file 1. Distribution of the control group. [file 12903_2023_2788_MOESM1_ESM.docx]

**Additional file 1.** Distribution of the control group.

|  |  | Sex | | Age (adolescence) | | |
| --- | --- | --- | --- | --- | --- | --- |
|  |  |  | | Early | Middle | Late |
| Variables | | Male | Female | 10 to <14 | 14 to <17 | 17 to <20 |
| Sex | Male | 15 |  | 6 | 4 | 5 |
|  | Female |  | 15 | 4 | 6 | 5 |
| Age (adolescence) | Early | 6 | 4 |  |  |  |
|  | Middle | 4 | 6 |  |  |  |
|  | Late | 5 | 5 |  |  |  |
| Total (each) |  | 30 | | 10 | 10 | 10 |
