# Supplementary material for: Comprehensive positional and morphological assessments of the temporomandibular joint in adolescents with skeletal Class III malocclusion: a retrospective CBCT study
Source: BMC Oral Health. 2023 Feb 7;23:78. doi: 10.1186/s12903-023-02788-4 (PMC9903422; doi:10.1186/s12903-023-02788-4)
Supplement: Supplementary file 2 — Additional file 2. STROBE Statement—checklist of items that should be included in reports of observational studies. [file 12903_2023_2788_MOESM2_ESM.docx]

STROBE Statement—checklist of items that should be included in reports of observational studies

|  | **Item No.** | **Recommendation** | **Page No.** | **Relevant text from manuscript** |
| --- | --- | --- | --- | --- |
| **Title and abstract** | 1 | (*a*) Indicate the study’s design with a commonly used term in the title or the abstract | 1 | cross-sectional retrospective study |
|  |  | (*b*) Provide in the abstract an informative and balanced summary of what was done and what was found | 1 | This study aimed to use cone-beam computed tomography (CBCT) to evaluate the position and morphology of the temporomandibular joint (TMJ) in adolescents with skeletal Class III malocclusion.  There were significant differences in the condyle-fossa relationships in skeletal Class III adolescents according to age, sex, and vertical skeletal patterns, except for the sides of the TMJ. |
| **Introduction** |  |  |  |  |
| Background/rationale | 2 | Explain the scientific background and rationale for the investigation being reported | 3, 4, 5 | Given these inconsistent findings, additional investigations are required to clarify the effects of skeletal Class III malocclusion on TMJ morphology and position. |
| Objectives | 3 | State specific objectives, including any prespecified hypotheses | 5 | Accordingly, this study aimed to use CBCT to identify the positional and morphological properties of the TMJ in adolescents with skeletal Class III malocclusion considering side, sex, age, and vertical skeletal patterns. |
| **Methods** |  |  |  |  |
| Study design | 4 | Present key elements of study design early in the paper | 5 | a cross-sectional CBCT-based study |
| Setting | 5 | Describe the setting, locations, and relevant dates, including periods of recruitment, exposure,  follow-up, and data collection | 5 | Conducted at the Stomatological Hospital of Chongqing Medical University, China |
| Participants | 6 | (*a*) *Cohort study*—Give the eligibility criteria, and the sources and methods of selection of participants. Describe methods of follow-up  *Case-control study*—Give the eligibility criteria, and the sources and methods of case ascertainment and control selection. Give the rationale for the choice of cases and controls *Cross-sectional study*—Give the eligibility criteria, and the sources and methods of selection of  participants | 5, 6 | Conducted on individuals’ CBCT examinations and dental records from February 2018 to July 2021 |
|  |  | (*b*) *Cohort study*—For matched studies, give matching criteria and number of exposed and unexposed  *Case-control study*—For matched studies, give matching criteria and the number of controls per  case |  | Not applicable |
| Variables | 7 | Clearly define all outcomes, exposures, predictors, potential confounders, and effect modifiers.  Give diagnostic criteria, if applicable | 5, 6  Table 1  Additional file 1 |  |
| Data sources/  measurement | 8* | For each variable of interest, give sources of data and details of methods of assessment  (measurement). Describe comparability of assessment methods if there is more than one group | Figure 1, 2, 3, 4, 5 |  |
| Bias | 9 | Describe any efforts to address potential sources of bias | 9 | All measurements were taken by a single investigator with experience in evaluating TMJ regions. To determine intraexaminer reliability, each case was re-examined twice within a period of three weeks. Intraclass correlation coefficients of 0.97–1.00 were obtained, indicating that the measurements were reproducible. |
| Study size | 10 | Explain how the study size was arrived at | 5 | The sample size was calculated using G*Power software (Version 3.1, Franz Faul, Christian-Albrechts-Universitat, Kiel, Germany) according to Chen et al. [17], who demonstrated a difference in inclination of articular tubercle among three classes of vertical skeletal patterns in skeletal Class III malocclusion: hyperdivergent (37.48°±1.44°), normodivergent (36.02°±6.53°), and hypodivergent (46.65°±8.44°). The minimum sample size required to identify a difference among groups using analysis of variance (ANOVA) was 25 images for each subgroup, with a power of 90% and a significance level of 5%. |
| Quantitative variables | 11 | Explain how quantitative variables were handled in the analyses. If applicable, describe which groupings were chosen and why | 7, 8, 9  Figure 1,2,3,4,5 | SS, AS, PS, AIC, PIC, MIC, LIC, LAC, MAC, HF, WF, AEH, AEI and anteroposterior condylar position were used as continuous variables. |
| Statistical methods | 12 | (*a*) Describe all statistical methods, including those used to control for confounding | 9, 10 | All measurements were taken by a single investigator with experience in evaluating TMJ regions. To determine intraexaminer reliability, each case was re-examined twice within a period of three weeks. Intraclass correlation coefficients of 0.97–1.00 were obtained, indicating that the measurements were reproducible.  The mean and standard deviation of each variable were analyzed and presented as descriptive statistics. The Kolmogorov-Smirnov test showed that all data followed a normal distribution. For intergroup analyses, we compared the joint measurements between the controls and skeletal Class III malocclusion with different vertical skeletal patterns using an independent sample t-test. For intra-group comparisons, an independent sample t-test, an analysis of variance, and a paired t-test were performed to compare the features of the mandibular condyle and articular fossa in the skeletal Class III participants based on sex, age, side, and vertical skeletal patterns. Pearson correlation analysis was used to determine the correlation between the ANB or FH-GoGn of skeletal Class III adolescents and measurement items relating to condyle-fossa relationships. Percentages of shapes were assessed according to different vertical skeletal patterns. The IBM SPSS Statistics software was used to analyze all data. |
|  |  | (b) Describe any methods used to examine subgroups and interactions | 10 | For intra-group comparisons, an independent sample t-test, an analysis of variance, and a paired t-test were performed to compare the features of the mandibular condyle and articular fossa in the skeletal Class III participants based on sex, age, side, and vertical skeletal patterns. |
|  |  | (c) Explain how missing data were addressed | Not applicable |  |
|  |  | (d)*Cohort study*—If applicable, explain how loss to follow-up was addressed  *Case-control study*—If applicable, explain how matching of cases and controls was addressed *Cross-sectional study*—If applicable, describe analytical methods taking account of sampling | Not applicable |  |
|  |  | (e) Describe any sensitivity analyses | Not applicable |  |
| **Results** |  |  |  |  |
| Participants | 13* | (a) Report numbers of individuals at each stage of study – eg numbers potentially eligible, examined for eligibility, confirmed eligible, included in the study, completing follow-up, and analysed | 6, 7  Table 1  Additional file 1 | The study included 90 adolescents with skeletal Class III malocclusion (age: 15.04±2.89 years) and 30 controls (age: 14.83±3.05 years). |
|  |  | (b) Give reasons for non-participation at each stage | Not applicable |  |
|  |  | (c) Consider use of a flow diagram | Not applicable |  |
| Descriptive data | 14* | (a) Give characteristics of study participants (eg, demographic, clinical, social) and information on exposure and potential confounders. | Table 1 |  |
|  |  | (b) Indicate number of participants with missing data for each variable of interest | Not applicable |  |
|  |  | (c) *Cohort stud*y-Summarise follow-up time (eg, average and total amount) | Not applicable |  |
| Outcome data* | 15* | *Cohort study*—Report numbers of outcome events or summary measures over time | Not applicable |  |
|  |  | *Case-control study—*Report numbers in each exposure category, or summary measures of exposure | Not applicable |  |
|  |  | *Cross-sectional study—*Report numbers of outcome events or summary measures | Table 2, 3, 4, 5, 6 |  |
| Main results | 16 | (a) Give unadjusted estimates and, if applicable, confounder-adjusted estimates and their precision (eg, 95% confidence interval). Make clear which continuous variables were categorized. | Table 2, 3, 4, 5, 6 |  |
|  |  | (b) Report category boundaries when continuous variables were categorized | Table 2, 3, 4, 5, 6 |  |
|  |  | (c) If relevant, consider translating estimates of relative risk into absolute risk for a meaningful time period. | Not applicable |  |
| Other analyses | 17 | Report other analyses done—eg analyses of subgroups and interactions, and sensitivity analyses | Table 3, 4, 5 |  |
| **Discussion** |  |  |  |  |
| Key results | 18 | Summarise key results with reference to study objective | 13-20 | In conclusion, there were significant differences in the condyle-fossa relationships between different ages, sexes, and vertical skeletal patterns, but not between the left and right sides of the TMJ, in adolescents with skeletal Class III malocclusion. This finding can be used clinically and radiographically to assess the condyle and glenoid fossa comprehensive features in adolescents with skeletal Class III malocclusion, hence providing a basis for better TMD diagnosis and orthodontic treatment. |
| Limitations | 19 | Discuss limitations of the study, taking into account sources of potential bias or imprecision. Discuss  both direction and magnitude of any potential bias | 20 | In addition to its strengths, this study has a few drawbacks. Although the sample size was predetermined in our research, additional investigations involving a greater number of participants are required to validate the current findings. To overcome study design limitations, a more precise TMJ analysis, including measurements of mandibular condylar motion, occlusal force, and stress direction at the TMJ, should be performed. |
| Interpretation | 20 | Give a cautious overall interpretation of results considering objectives, limitations, multiplicity of analyses, results from similar studies, and other relevant evidence | 13-20 | Different muscular forces act on the condyle-fossa complex, resulting in various TMJ remodeling |
| Generalisability | 21 | Discuss the generalisability (external validity) of the study results | 13 | In adolescents with skeletal Class III malocclusion, evaluating TMJ characteristics may help dental professionals detect radiographic abnormalities, allowing for better treatment planning. Consequently, accurate measurements of these imaging values in conjunction with clinical examinations may be highly essential for the diagnosis and treatment of skeletal Class III malocclusion. |
| **Other information** |  |  |  |  |
| Funding | 22 | Give the source of funding and the role of the funders for the present study and, if applicable, for the  original study on which the present article is based | 23, 24 | This study was supported by the Program for the Science and Technology Plan of Yubei District, Chongqing [No. 2022 (agriculture and social) 36] and Chongqing Science and Health Joint Medical Research Project [No. 2021MSXM114]. |

*Give information separately for exposed and unexposed groups.

Note: An Explanation and Elaboration article discusses each checklist item and gives methodological background and published examples of transparent reporting. The STROBE checklist is best used in conjunction with this article (freely available on the Web sites of PLoS Medicine at http://www.plosmedicine.org/, Annals of Internal Medicine at http://www.annals.org/, and Epidemiology at http://www.epidem.com/). Information on the STROBE Initiative is available at www.strobe-statement.org.
